# KSHV encoded LANA recruits Nucleosome Assembly Protein NAP1L1 for regulating viral DNA replication and transcription

**DOI:** 10.1038/srep32633

**Published:** 2016-09-07

**Authors:** Namrata Gupta, Suhani Thakker, Subhash C. Verma

**Affiliations:** 1 Department of Microbiology and Immunology, School of Medicine, University of Nevada, Reno, NV 89557, USA.

## Abstract

The establishment of latency is an essential for lifelong persistence and pathogenesis of Kaposi’s sarcoma-associated herpesvirus (KSHV). Latency-associated nuclear antigen (LANA) is the most abundantly expressed protein during latency and is important for viral genome replication and transcription. Replication-coupled nucleosome assembly is a major step in packaging the newly synthesized DNA into chromatin, but the mechanism of KSHV genome chromatinization post-replication is not understood. Here, we show that nucleosome assembly protein 1-like protein 1 (NAP1L1) associates with LANA. Our binding assays revealed an association of LANA with NAP1L1 in KSHV-infected cells, which binds through its amino terminal domain. Association of these proteins confirmed their localization in specific nuclear compartments of the infected cells. Chromatin immunoprecipitation assays from NAP1L1-depleted cells showed LANA-mediated recruitment of NAP1L1 at the terminal repeat (TR) region of the viral genome. Presence of NAP1L1 stimulated LANA-mediated DNA replication and persistence of a TR-containing plasmid. Depletion of NAP1L1 led to a reduced nucleosome positioning on the viral genome. Furthermore, depletion of NAP1L1 increased the transcription of viral lytic genes and overexpression decreased the promoter activities of LANA-regulated genes. These results confirmed that LANA recruitment of NAP1L1 helps in assembling nucleosome for the chromatinization of newly synthesized viral DNA.

Kaposi’s sarcoma-associated herpesvirus (KSHV), also referred to as human herpesvirus 8 (HHV8), is linked to Kaposi’s sarcoma, primary effusion lymphomas (PELs), and multicentric Castleman’s disease, which causes tumors in AIDS patients^1^,^2^. Like other herpesviruses, KSHV displays two distinct life cycles, the default latent and the productive lytic phase, and persists predominantly in the latent form. During latency, only a limited number of viral proteins are expressed, including the latency-associated nuclear antigen encoded by open reading frame 73 (ORF73)^3^,^4^. LANA is expressed in all KSHV-positive tissues and cell lines^5^. The full repertoire of viral gene expression occurs during the lytic replication (reactivation) phase, which is likely essential to maintaining the population of newly infected cells and the induction of viral pathogenesis^6^. LANA is a nuclear protein with 1162 amino acids and is 220–230 kDa in size. It interacts with various cellular and viral proteins to regulate transcription, cellular signaling, viral DNA replication, and genome maintenance^3^. During latent infection, KSHV DNA persists as multi-copy, chromatinized closed circular episomes tethered to the host chromosomes^3^,^7^. LANA binds to the viral genome in the TR region through its carboxyl terminus and attaches to the nucleosomes through the amino terminus for efficient persistence^8^,^9^.

LANA, a multifunctional protein, also plays a role in maintaining viral latency, efficient segregation of episomal DNA, and oncogenesis^3^,^10^. LANA has also been shown to modulate the transcription of a variety of cellular and viral promoters^11^,^12^. LANA can serve both as an activator and repressor of gene transcription, suppressing p53 and VHL-driven transcriptions and activating the transcriptions of E2F1 and cyclin-dependent kinase-2 (CDK2)^3^,^13^,^14^. LANA has been implicated in tumorigenesis through its interactions and interference with cellular pathways associated with cell cycle control, apoptosis, gene expression and immune regulation^15^,^16^. Furthermore, LANA negatively regulates the transcription of viral lytic genes during the establishment of latency^17^. It also represses the transcription of RTA, an immediate early gene encoded by ORF50, which activates the switch from latency to lytic replication^18^,^19^. Recent studies have demonstrated that LANA-recruited KAP1 (Krüppel-associated box domain-associated protein 1), a transcriptional repressor, plays a critical role in the silencing of a lytic gene’s expression to facilitate the establishment of latency^20^.

In addition to modulating gene transcription, LANA recruits a number of proteins to regulate KSHV DNA replication and segregation of the newly synthesized genome to the progeny nuclei^21^,^22^. LANA mediates KSHV TR DNA replication through its recruitment of host-cellular machinery, including origin recognition complexes (ORCs) and minichromosome maintenance (MCMs) proteins^23^,^24^. LANA’s binding to the TR element is necessary for TR-mediated DNA replication^23^,^24^. LANA achieves this with the help of cellular proteins, including chromatin-associated proteins and proteins involved in DNA replication^25^,^26^. Additionally, LANA interacts with Bub1 that recruits PCNA (proliferating cell nuclear antigen) to the KSHV genome to mediate latent replication of KSHV episomes in the infected cells^27^,^28^. These processes occur in synchrony with the host cells^3^,^11^. Various approaches have been taken to gain a better understanding of how LANA achieves these activities. Protein-protein interaction studies have identified a large number of LANA binding proteins that are involved in cell cycle regulation, tumor progression and epigenetic control^10^,^29^.

NAP1L1 is a member of the nucleosome assembly protein 1 (NAP1) which carries out a number of roles related to transcription and DNA replication^30^,^31^. Mammalian cells possess five members of the NAP1 family of proteins^32^; of these, three are expressed exclusively in neurons^33^,^34^ and the other two, referred to as NAP1L1 and NAP1L4, are ubiquitously expressed^31^,^35^. The NAP1 family of proteins is characterized by the presence of a highly conserved central domain (the NAP domain) and acidic C-terminal sequences^36^. The NAP domain is necessary for histone binding and nucleosome assembly^37^. Subsequent studies have demonstrated that nucleosome assembly protein associates with histone, H2A-H2B dimers in human cells^38^, and *Drosophila* embryo extracts^39^ to assemble the nucleosome, thus suggesting their role in *de novo* chromatin assembly^38^. Furthermore, NAP1 has the ability to facilitate the assembly of nucleosomes *in vitro*^40^ and has also been implicated in several other processes, including cell-cycle regulation^41^, transcriptional activation through chromatin remodeling^42^, and nucleosome sliding^43^. Nucleosome assembly proteins have also been reported to interact with p300/CBP and acetyltransferases^42^ and can function in the nucleocytoplasmic shuttling of histones between the cytoplasm and the nucleus during cell cycle progression^44^.

In this study, we determined that LANA binds to NAP1L1 in the KSHV-infected cells. The binding domain of LANA with NAP1L1 was mapped to the amino-terminal domain adjacent to the chromosome-binding domain (CBD). These two proteins co-localized primarily in the G_1_/S-phase of the cell cycle. Since the nucleosome assembly proteins associate with histone modification and nucleosome remodeling factors to influence DNA replication and transcription, we hypothesize that LANA recruits these factors for replication and transcriptional activities. We demonstrate that LANA recruits NAP1L1 to the KSHV genome and plays a critical role in regulating the expression of viral genes to facilitate the establishment of KSHV latency. We also determined the functional significance of these interactions and the role of nucleosome assembly protein in chromatin assembly using biochemical assays, such as micrococcal nuclease and chromatin immunoprecipitation assays, which confirmed their role in assembling the nucleosome on the viral DNA. Conclusively, these results reveal the importance of nucleosome assembly protein in LANA-mediated DNA replication and replication-coupled nucleosome assembly.

## Results

### LANA interacts with NAP1L1 in KSHV-infected cells

**Figure 1:**
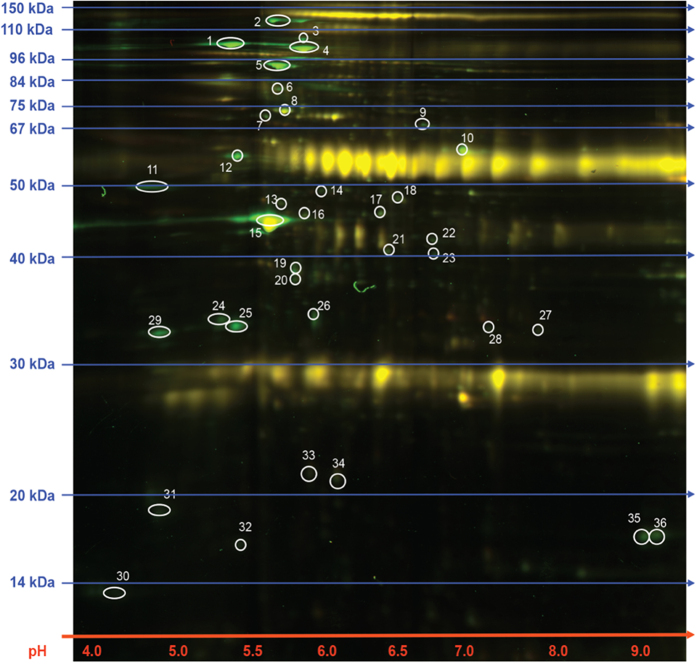
2D-DIGE and proteomic analysis for the identification of LANA binding proteins. Immunoprecipitated proteins with anti-LANA and control IgG antibodies were labeled with dyes, Cy3 and Cy5, respectively before separating by IEF and SDS-PAGE. Representative image of the 2D-DIGE analyses showing superimposed Cy3 and Cy5 emissions. The differential proteins spots were subjected to MALDI-TOF-MS analysis.

**Figure 2:**
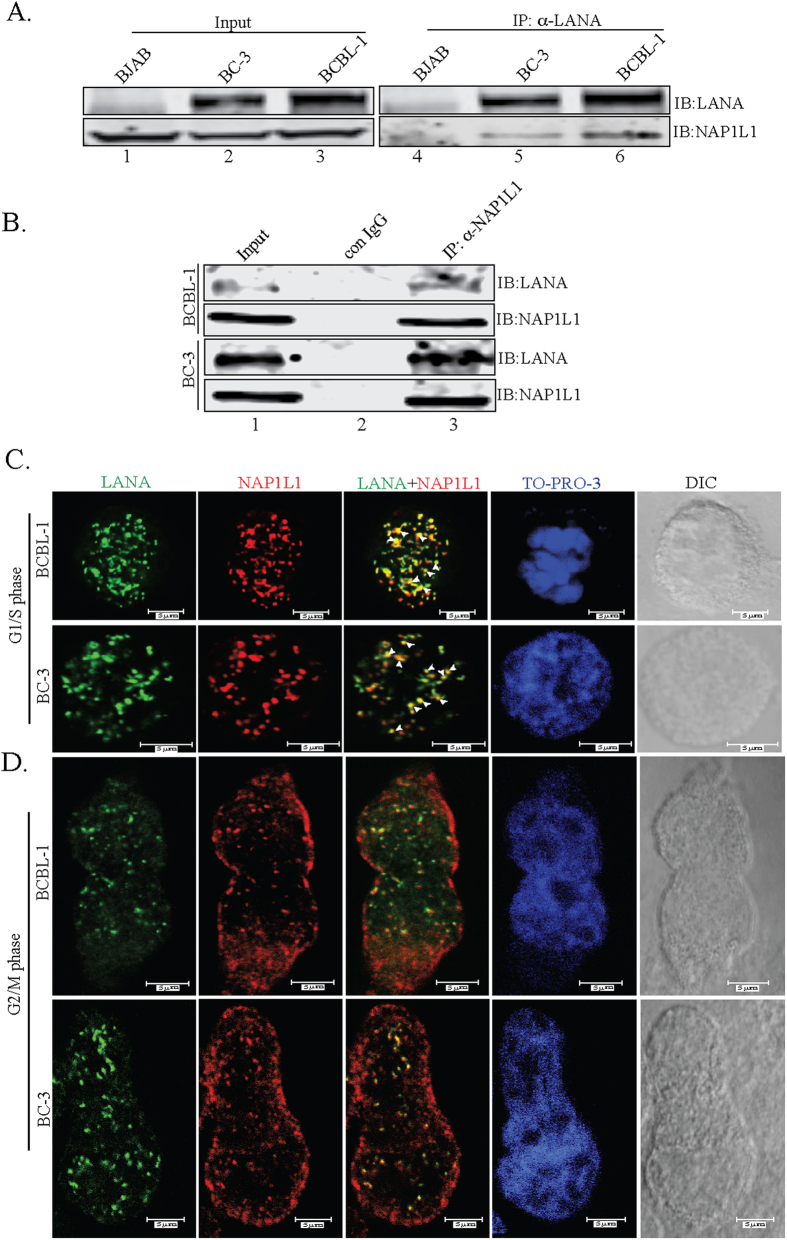
LANA associated and co-localized with NAP1L1 in KSHV-positive cells. **(A)** Co-immunoprecipitation of endogenous LANA with nucleosome assembly protein, NAP1L1 from BC-3 and BCBL-1 cells. LANA was immunoprecipitated by using mouse anti-LANA antibody from precleared cellular lysates of BC-3 or BCBL-1 cells. KSHV-negative cells, BJAB were used as control. Immunobloting with anti-NAP1L1 shows specific binding in BC-3 and BCBL-1 cells (lanes, 5 and 6). **(B)** Immunoprecipitation with anti-NAP1L1 antibody and subsequent detection of LANA shows specific association in BCBL-1 and BC-3 cells (lane 3). An isogenic antibody, mouse IgG (con IgG) was used as a control. **(C)** Immunofluorescence analysis of endogenous LANA and NAP1L1 in BCBL-1 and BC-3 cells. Cells were stained with rat anti-LANA and rabbit anti-NAP1L1 antibodies and subsequently detected with anti-rat Alexa Fluor 488 (LANA) and anti-rabbit Alexa Fluor 594 (NAP1L1). Nuclei stained with TO-PRO-3 is shown in blue. LANA and NAP1L1 colocalize in the nuclear compartments as detected by yellow dots. DIC images were captured to show the cell morphology. Colocalizing signals are indicated as white arrowheads in the merge (LANA + NAP1L1) panels. Magnification bars show 5 μm. **(D)** Immunofluorescence analysis in G_2_/M arrested cells. G_2_/M arrested cells were used for the localization of NAP1L1 and LANA proteins using specific antibodies and detected with Alexa Fluor 488 (LANA) and anti-rabbit Alexa Fluor 594 (NAP1L1). Magnification bars show 5 μm.

A number of LANA binding proteins were identified by 2D-DIGE, which included previously identified proteins as well as additional molecule including a highly conserved nucleosome assembly protein, NAP1L1 (Fig. 1 and Table 1). Since LANA is required for TR-mediated replication, we hypothesized that recruitment of NAP1L1 may be important for the assembly of nucleosomes on the newly replicated DNA. To determine the interactions of nucleosome assembly protein NAP1L1 with LANA in KSHV-infected primary effusion lymphoma (PEL) cells, a co-immunoprecipitation (co-IP) assay was performed on the endogenous proteins from KSHV-positive, BCBL-1 and BC-3 cells. Immunoprecipitation (IP) with anti-LANA antibody and detection of NAP1L1 showed that LANA precipitated NAP1L1 from the KSHV-positive cells (Fig. 2A, lanes 5 and 6). The lack of NAP1L1 protein from BJAB cells with anti-LANA antibody confirmed the specificity of LANA’s association with NAP1L1 (Fig. 2A, lane 4). To further analyze the specificity of this interaction, a reverse co-IP assay was performed using anti-NAP1L1 antibodies from BCBL-1 and BC-3 cell lines. Immunoprecipitation and subsequent detection with anti-LANA antibody showed that NAP1L1 efficiently precipitates LANA from these KSHV-positive BCBL-1 and BC-3 cells (Fig. 2B, lane 3). These results confirmed that NAP1L1 forms a complex with LANA in KSHV-infected cells.

In order to further validate the interactions between LANA and NAP1L1 and to determine whether these proteins are in the same nuclear compartment, we examined colocalization of LANA with NAP1L1 during the replicating phase, G_1_/S of BCBL-1 and BC-3 cells. As expected, LANA showed a typical punctate staining pattern in the nuclei of the PEL cells (Fig. 2C, panels in green) and NAP1L1 (Fig. 2C, panels in red) was also detected as a distinct pattern in the same nuclear compartment as LANA, thus suggesting a colocalization of these two proteins (Fig. 2C, detected as a yellow signal in the LANA + NAP1L1 panels). Nuclei stained with TO-PRO-3 showed that the colocalization signals were in the nuclei of these cells and the differential interference contrast (DIC) showed the integrity of the cells (Fig. 2C). Detection of these two proteins in the same nuclear compartments suggested that LANA has a role in regulating the nucleosome structure of the viral genome during replication.

It has been well established that NAPs localize to the nuclei during the S-phase and become predominantly cytosolic during the G_2_ phase in *Drosophila* embryos^39^. Based on the G_1_/S dependent nuclear localization of NAP1, we next examined the localization of LANA and NAP1L1 proteins in PEL cells arrested in the G_2_/M phase by treating them with colchicine. We found that NAP1L1 localized mainly in the cytoplasm with very low amounts in the nuclei, as expected (Fig. 2D). Detection of LANA and NAP1L1 in these cells showed a significant decrease in the colocalization patterns suggesting that these two proteins primarily associate during the replication phase of the cells for modulating the chromatin structure.

### NAP1L1 interacts with the amino-terminus of LANA through its acidic domain

**Figure 3:**
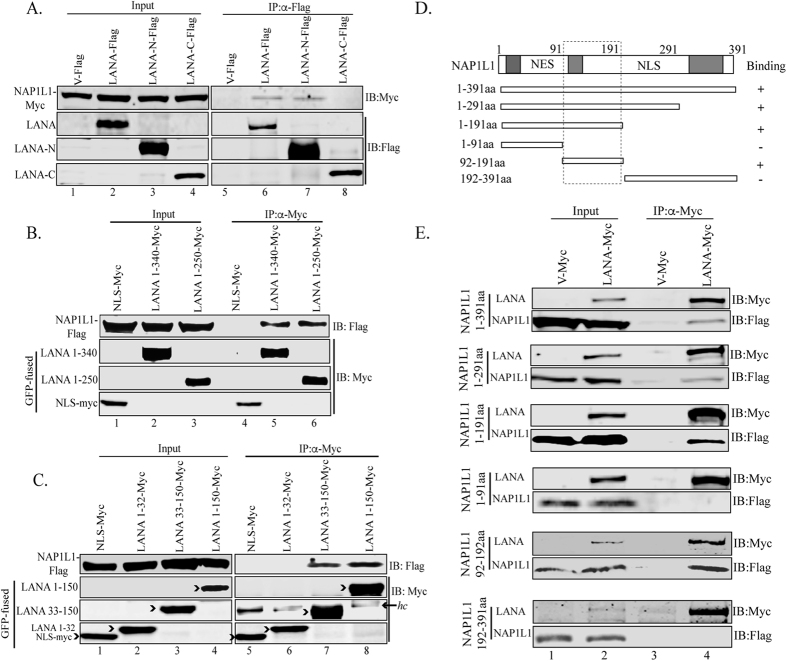
The amino terminus of LANA interacted with NAP1L1 in the acidic domain. **(A)** HEK 293T cells were transfected with Flag-tagged pA3F empty vector, pA3F LANA, pA3F LANA-N and pA3F LANA-C along with Myc-tagged NAP1L1. The lysate was subjected for immunoprecipitation with anti-Flag antibody followed by detection with anti-Myc antibody, which shows binding with full length and the amino terminal domain (lanes 6 and, 7) but not with control (lane 5) or carboxy terminal domain (lane 8). **(B**,**C)** HEK 293T cells were transfected with GFP-NLS-Myc (empty vector), LANA truncation spanning aa 1 to 340 (LANA 1-340)-GFP-Myc, LANA 1-250-GFP-Myc, LANA 1-150-GFP-Myc, LANA 33-150-GFP-Myc, or LANA 1-32-GFP-Myc along with Flag-tagged NAP1L1. Cell lysates were subjected to IP with anti-Myc antibody to detect co-immunoprecipiated NAP1L1. LANA aa 1-340, aa-1-250, 1-150 and 33-150 bound to NAP1L1. *hc* represents the heavy chain of the antibody. **(D)** Schematic of NAP1L1 truncation domains and its binding summary to LANA. The acidic domains of the protein are shown by grey boxes and the NES and NLS are the nuclear export and nuclear localization signals, respectively. **(E)** HEK 293T cells were cotransfected with pA3M empty vector, pA3M LANA along with Flag-tagged NAP1L1 aa 1 to 391, NAP1L1 aa 1 to 291, NAP1L1 aa 1 to 191, NAP1L1 aa 1 to 91, NAP1L1 aa 92 to 191 and NAP1L1 aa 192 to 391. The cell lysates were immunoprecipitated with anti-Myc antibody to detect the NAP1L1 truncations with anti-Flag antibody (lane 4 in each panel).

Next, we wanted to determine the domains of LANA responsible for NAP1L1 interaction. We performed co-IP assays in HEK 293T cells transfected with either the vector control, full-length LANA, LANA-N (1 to 340 aa), or LANA-C (940 to 1162 aa) tagged with Flag epitope, along with a Myc-tagged full-length NAP1L1 vector. Immunoprecipitation with anti-Flag antibody and subsequent detection of NAP1L1 using an anti-Myc antibody showed co-precipitation of NAP1L1 with full-length LANA as well as its N-terminus but not with the C-terminus of LANA (Fig. 3A, lanes 6, 7 and 8). Empty Flag vector with NAP1L1 did not show any precipitation of NAP1L1, confirmed the specificity of the interaction (Fig. 3A, lane 5). We confirmed that NAP1L1 directly binds to the amino terminal domain of LANA (LANA-N) by performing an *in-vitro* binding assay with bacterially expressed GST-LANA-N and *in-vitro* translated NAP1L1, which showed specific association of LANA-N fused to GST but not with GST alone (data not shown). Further, we wanted to narrow down the domains of LANA responsible for NAP1L1 interaction. For this, LANA truncations spanning amino acids 1–340 and 1–250 tagged with Myc epitope were co-expressed with full-length Flag-tagged NAP1L1 in HEK 293T cells for co-IP assay. Detection of NAP1L1 with both truncations confirmed their binding (Fig. 3B, lanes 5 and 6). We further used smaller truncations of LANA spanning amino acids, 1–150, 1–32 and 33–150 tagged with Myc epitope in binding assay with NAP1L1. Detection of NAP1L1 in co-precipitating lanes confirmed binding of LANA 1–150 aa and 33–150 aa but not 1–32 aa (Fig. 3C, compare lanes 7 and 8 with 6). Vector control, GFP-NLS-Myc, did not show precipitation of NAP1L1, suggesting specific association of LANA truncation mutants (Fig. 3B, lane 4 and 3C, lane 5). Interestingly, LANA 1–32 aa has the chromatin-binding domain (CBD)^9^ and NAP1L1 binds adjacent to the CBD.

Furthermore, we wanted to determine the domains of NAP1L1 responsible for interaction with LANA. To this end, we generated truncation mutants of NAP1L1 containing amino acids 1–391, 1–291, 1–191, 1–91, 92–191 and 192–391 tagged with Flag epitope (Fig. 3D). These truncations were co-expressed with full-length LANA tagged with Myc epitope or with empty Myc vector in HEK 293T cells. Immunoprecipitation analysis with anti-Myc antibody and subsequent detection of NAP1L1 truncations with anti-Flag antibody showed binding of NAP1L1 full-length (1–391 aa) and 1–291 aa and 1–191 aa truncations (Fig. 3E, lane 4). LANA was unable to precipitate NAP1L1 mutants spanning 1–91 aa and 192–391 aa but precipitated 92–191 aa, suggesting that LANA binding to NAP1L1 lies in the acidic domains of NAP1L1 (Fig. 3E, lane 4). Overall, these results suggest that NAP1L1 domain interacting with LANA protein is localized to amino acids 92–192, which is an acidic domain.

### Nucleosome assembly protein, NAP1L1 is required for LANA-dependent DNA replication

**Figure 4:**
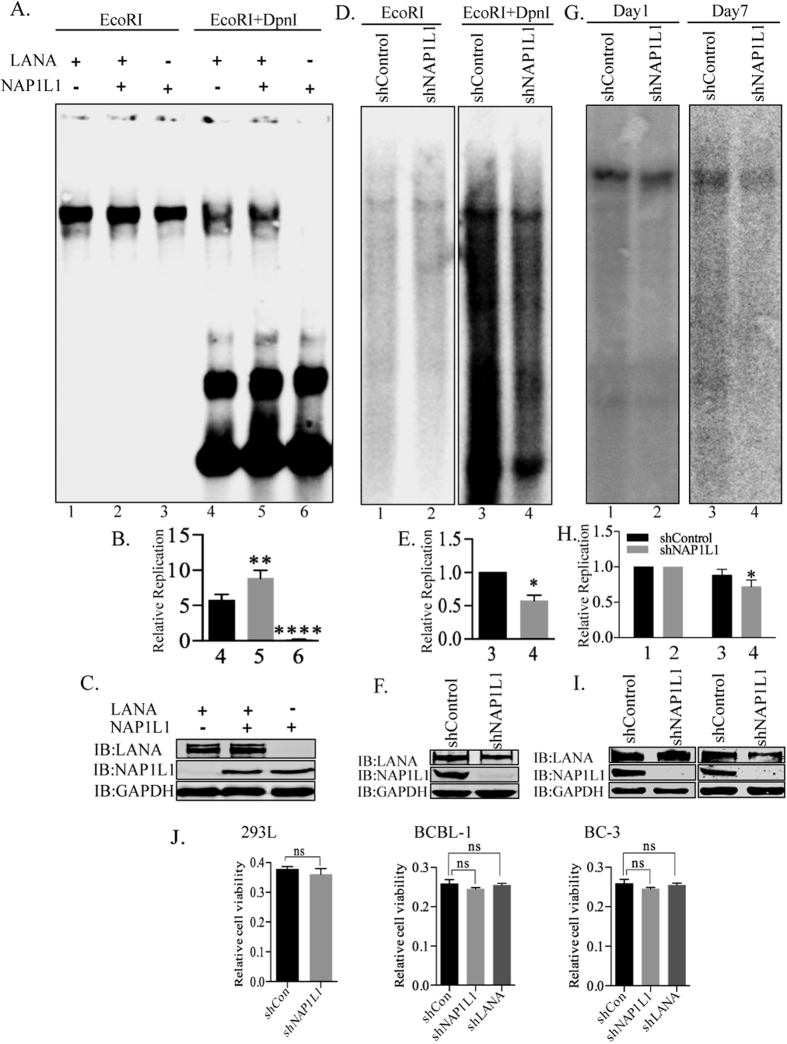
NAP1L1 regulated viral DNA replication. **(A)** HEK 293L cells were transfected with plasmids expressing NAP1L1 along with TR plasmid in presence of LANA expressing vector (pA3F-LANA). Extracted plasmids were harvested and digested with either *Eco*RI (to linearize) or with *Eco*RI and *Dpn*I. Cells expressing NAP1L1 show a pronounced increased in *Dpn*I-resistant replicated DNA (lane 5) compared to the cells without NAP1L1 expression (lane 4), Cells lacking LANA did not show any *Dpn*I-resistant band, as expected (lane 6). **(B)** Densitometric analysis of the *Dpn*I resistant bands compared to the respective input lanes (10% of the DNA digested with EcoRI) and plotted using Prism 6.0 software. *P* values of <0.01 (**) and <0.0001 (****) are indicated. **(C)** Western blots showing expression of LANA, NAP1L1 and GAPDH. **(D)** HEK 293L cells depleted for NAP1L1 (shNAP1L1) and the control cells (shControl) were transfected with TR plasmid along with LANA. Extracted DNA was digested with either *Eco*RI (to linearize) or with *Eco*RI and *Dpn*I to determine the levels of replication. **(E)** Densitometric analysis of the *Dpn*I resistant bands compared to the respective input lanes and plotted using Prism 6.0 software. *P* value of <0.05 (*) is indicated. **(F)** Expression of NAP1L1 and GAPDH in the cells used for replication assays. **(G)** NAP1L1 depleted (shNAP1L1) HEK293L cells were transfected with TR plasmid along with LANA expressing vector. Parts of the transfected cells were harvested at Day 1 and remaining was allowed to grow for a week to evaluate the plasmid maintenance in cells lacking NAP1L1. Extracted DNA was digested with *Eco*RI Southern probed to detect the TR plasmid. **(H)** Amounts of TR plasmid maintained for 7 days in shNAP1L1 cells were compared to the shControl cells by determining the relative band intensities of shNAP1L1 and shControl cells at day 1 and plotted using Prism 6.0 software. *P* value of <0.05 (*) is indicated. All the experiments were repeated at least three times. NAP1L1 depleted cells shows slight reduction in the maintenance of the TR plasmid. **(J)** Cell viability analysis by MTT assay of HEK293L cells depleted for NAP1L1. Cell viability of KSHV infected (BCBL-1 and BC-3) depleted for NAP1L1 and LANA. Data shown are the means of three separate experiments. Statistical analysis was performed using an unpaired t-test for the significance (p-value). n.s. not statistically significant.

Binding of LANA to the TR is known to activate DNA replication and regulate transcription of gene promoters bound by LANA, including RTA and K1 genes^12^,^19^. Both of these processes are likely to be regulated by nucleosome positioning and histone modifications, and the recruitment of nucleosome assembly factors by LANA postulated its role in replication and transcription. In order to determine the roles of NAP1L1 in replication activity, transient replication was performed by transfecting the TR plasmid with NAP1L1 and LANA-expressing vectors. Replication was assayed at 4 days post-transfection by digesting the extracted DNA with *Dpn*I, which yields a *Dpn*I-resistant band after replication. Extracted DNA was digested with either *Eco*RI to linearize, or with *Eco*RI and *Dpn*I to digest, the unreplicated plasmids, followed by detection of replicated DNA in a Southern blot using a ^32^P-labeled TR probe. Data showed that expression of NAP1L1 increased the amounts of replicated DNA when compared to the LANA expressing cells (Fig. 4A, compare lanes 4 and 5). The relative band intensities of the *Dpn*I-resistant bands were calculated based on their respective inputs, which showed an increase in the replication in NAP1L1-expressing cells (Fig. 4B, compare bars 4 and 5). Cells lacking LANA expression did not show any *Dpn*I-resistant bands, as expected (Fig. 4A and B, lane 6). The quantitation of band intensities was averaged from three independent experiments (Fig. 4B). The levels of LANA and NAP1L1 were determined in a fraction of cells used for DNA extraction for *Dpn*I sensitivity assay (Fig. 4C).

Additionally, we performed transient replication assays in HEK 293L cells depleted with the nucleosome assembly protein, NAP1L1, by stably expressing a control (shControl) or a specific shRNA for NAP1L1 (shNAP1L1) through lentiviral vectors. TR containing plasmid and LANA-expression vector transfected cells (shControl and shNAP1L1) were harvested at 96 h post-transfection for DNA extraction. The DNA was digested with either *Eco*RI or *Dpn*I and *Eco*RI to detect the replicated plasmid using a ^32^P-labeled TR probe in a Southern blot. As shown in Fig. 4D and in the relative quantitation of the *Dpn*I-resistant band (Fig. 4E), compared to their inputs in the *Eco*RI lanes, NAP1L1-depleted cells showed reduced replication (Fig. 4D,E, lanes 3 and 4). These results suggest that NAP1L1 regulates LANA-mediated replication, possibly by assembling the nucleosome on the newly synthesized DNA post-replication. The levels of NAP1L1 were detected by anti-NAP1L1 antibody, which showed efficient reduction in specific shRNA cells (Fig. 4F). Expression of LANA was detected with anti-Flag antibody (Fig. 4F).

Since NAP1L1 helps in assembling the nucleosome on the replicating DNA, we wanted to determine the consequence of NAP1L1 depletion on the maintenance of TR-containing plasmid by determining the plasmid levels seven days post-infection. TR-containing plasmid and LANA expression vectors were transfected in NAP1L1-depleted or control HEK 293L cells. Transfected DNA was recovered from part of the transfected cells at day one for the reference and day seven for determining the levels of maintained plasmid. The DNA was digested with *Eco*RI, which linearizes the TR-containing plasmid, resolved and subjected for Southern detection with TR probe (Fig. 4G). Intensities of TR-containing plasmid were determined by densitometry and the relative levels of TR plasmid at day seven were calculated by using day one as reference, which showed a slight reduction in NAP1L1-depleted cells (Fig. 4H). This suggested that NAP1L1 is required for assembling the nucleosome on replicating DNA for their maintenance. Western blots were performed to verify the depletion of NAP1L1 and the expression of LANA in these cells (Fig. 4I).

Nucleosome assembly proteins are involved in cell cycle progression and cell proliferation^45^, therefore, we wanted to determine whether depletion of NAP1L1 was affecting the cell viability of HEK293L cells in replication assay. We performed water-soluble tetrazolium salt (MTT) assay to compare the cell viability of NAP1L1 depleted (shNAP1L1) cells with the control (shCon) cells (Fig. 4J). The data shows that there were only slight reduction in cell growth and viability of NAP1L1 depleted cells (Fig. 4J). We also compared the cell viability of BCBL-1 and BC-3 cells depleted for NAP1L1 (shNAP1L1) with the control cells (shCon). Similarly, both the KSHV positive cell lines (BCBL-1 and BC-3) were not significantly affected in cell viability due to NAP1L1 depletion (Fig. 4J).

### NAP1L1 depletion reduced episomal DNA in BCBL-1 and BC-3 cells

**Figure 5:**
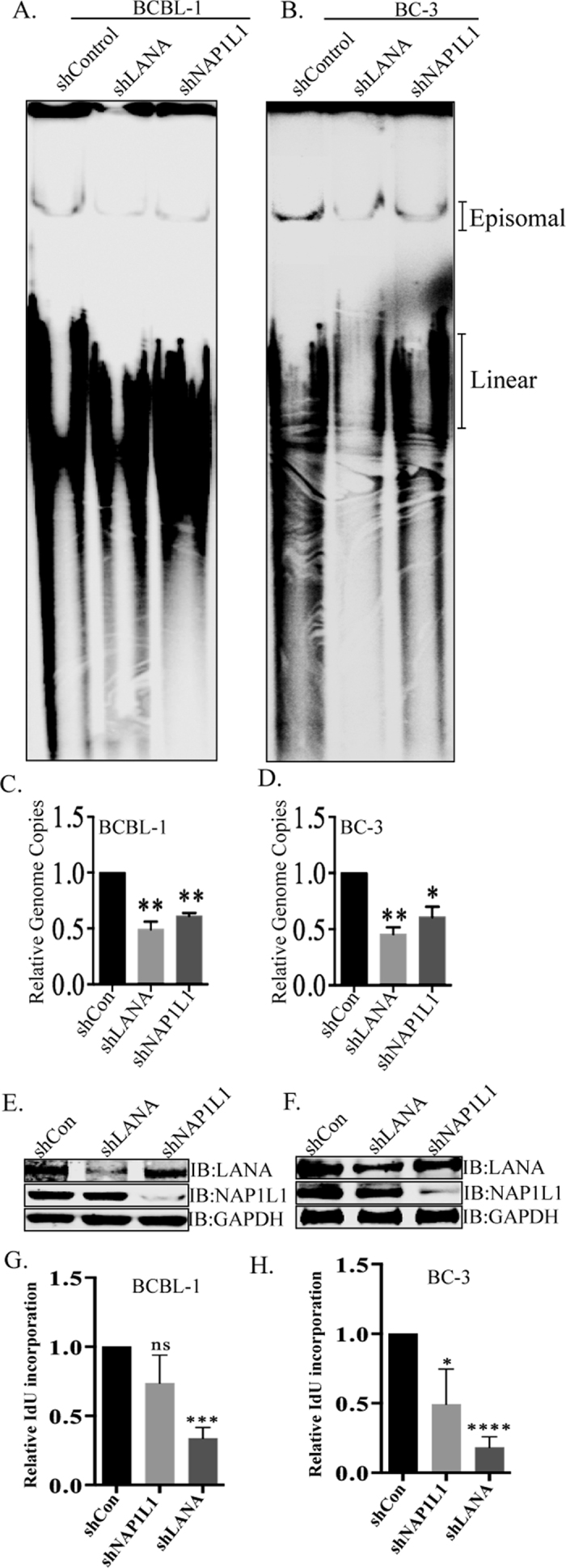
NAP1L1 depletion in BCBL-1 and BC-3 reduced persistence of KSHV episomes. **(A**,**B)** Gardella gel analysis assessing the presence of episomal and linear KSHV genomes in NAP1L1 depleted (shNAP1L1) BCBL-1 and BC-3 cells as compared to the control (shControl) cells. Approximately one million cells analyzed for the presence of episomal DNA in a Gardella gel. Episomal DNA bands have slower electrophoretic mobility as compared to the linear genome. NAP1L1 depleted cells showed lower levels of episomal genome as compared to the control. LANA depleted cells (shLANA) showed significantly reduced KSHV episomal copies as expected. **(C,D)** Relative KSHV genome copies were determined by quantitative real-time PCR from DNA extracted from the shControl, shLANA and shNAP1L1 cells using KSHV TR-specific primers, which showed reduction in KSHV genomes copies in NAP1L1 and LANA depleted cells. *P* values of <0.05 (*) and <0.01 (**) are indicated. Western blots showing expression level of LANA and NAP1L1 in LANA depleted, NAP1L1 depleted and control BCBL-1 **(E)** and BC-3 **(F)** cells. The GAPDH immunoblot shows equal loading of the cell lysates. **(G**,**H)** Viral DNA replication in NAP1L1 and LANA depleted cells using IdU incorporation in KSHV infected BCBL-1 **(G)** and BC-3 **(H)** cells. IdU incorporated viral DNA was immunoprecipitated with anti-IdU antibody for the determination of replicated DNA in a qRT-PCR at the TR region. Both shNAP1L1 and shLANA cells show reduction in IdU incorporated DNA as compared to the control cells. Data shown are the means of three independent experiments. The *P* values were calculated by two-tailed t tests using Prism 6.0 software at a significance level of *P* < 0.05 (*); *P* < 0.001 (***); *P* < 0.0001 (****) or n.s. (not statistically significant).

Since NAP1L1 depletion reduced the maintenance of TR-containing plasmids *ex-vivo*, we wanted to determine whether a depletion of NAP1L1 in KSHV-infected, BCBL-1 and BC-3 cells has an effect on the persistence of the KSHV genome. To this end, we depleted NAP1L1 by transducing lentiviral vectors containing shNAP1L1 or shControl. These cells were analyzed for the detection of KSHV episomes by Gardella gel analysis in a Southern blot (Fig. 5A,B). Interestingly, the band intensities of the episomal copies of the KSHV genomes were reduced in the NAP1L1-depleted, BCBL-1 and BC-3 cells as compared to the control cells (Fig. 5A,B). We also depleted LANA, which is critical for the maintenance of the KSHV episomes, and assayed the episomal copies along with the NAP1L1-depleted cells. The data showed a significant reduction in episomal copies of the KSHV genome, as expected (Fig. 5A,B). We also determined the total KSHV genome copies by extracting DNA using Hirt’s method and quantifying the KSHV genome in a real-time quantitative PCR assay (Fig. 5C,D). Both the BCBL-1 and BC-3 cells, depleted for NAP1L1, showed a reduction in KSHV genome copies when compared to the control cells (Fig. 5C,D). LANA depletion had a significant reduction in KSHV genome copies, as expected. This confirmed that NAP1L1 is important for maintaining the viral genome in the KSHV-infected cells. The levels of LANA and NAP1L1 were detected by anti-LANA and anti-NAP1L1 antibodies, which showed efficient reduction of target proteins in the indicated shRNAs transduced BCBL-1 and BC-3 cells (Fig. 5E,F).

Since the depletion of NAP1L1 and LANA significantly decreased KSHV genome copies in KSHV infected B-lymphoma cells, we wanted to determine the replication of viral genome by pulsing the cells with a thymidine analog, IdU and detecting the levels IdU incorporation on viral TR DNA. Imunoprecipitation of IdU labeled DNA with anti-IdU antibody and amplification of TR regions showed significant reduction of IdU labeled TR copies in LANA depleted cells, as expected, compared to the control cells (Fig. 5G,H). Not surprisingly, NAP1L1 depletion showed slight reduction in IdU incorporation confirming that NAP1L1 also contribute to the replication of TR region (Fig. 5G,H).

### LANA recruited the nucleosome assembly protein at the terminal repeats

**Figure 6:**
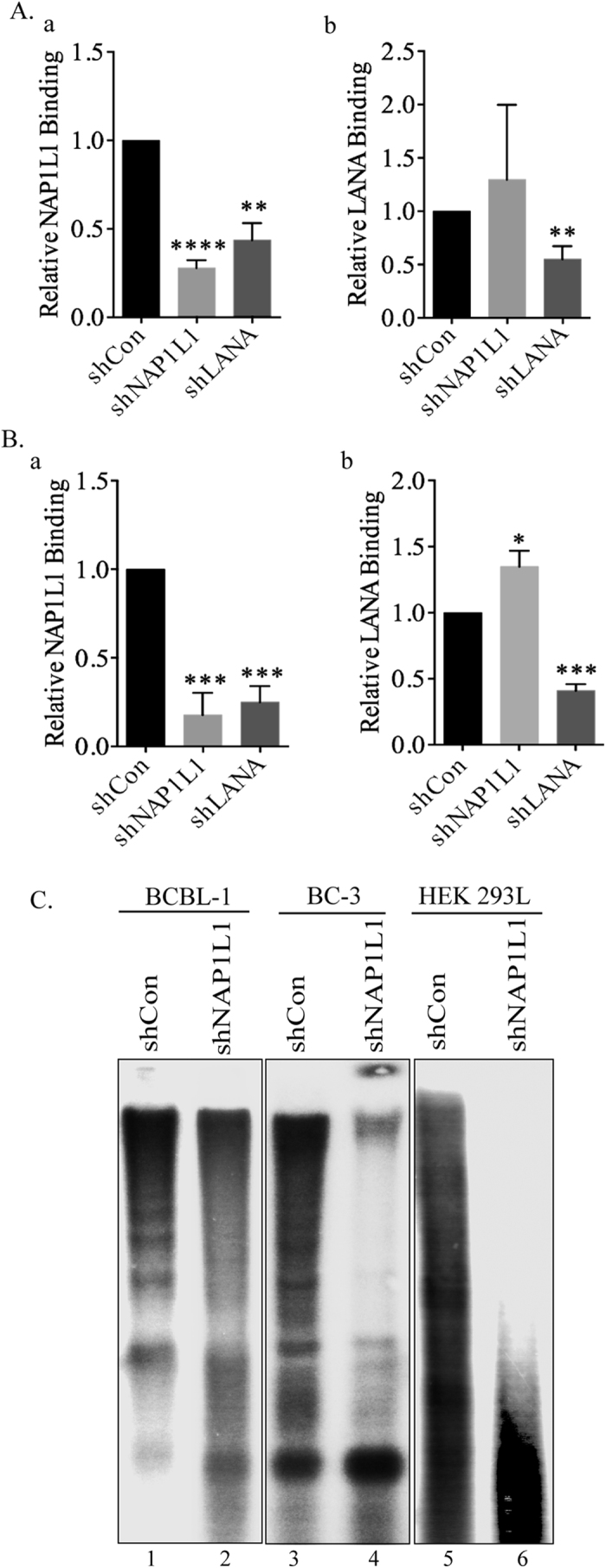
NAP1L1 associated with viral chromatin to control chromatin organization. Chromatin immunoprecipitation (ChIP) assays were performed from LANA and NAP1L1 depleted BCBL-1 **(A)** and BC-3 **(B)** cells with anti-LANA and anti-NAP1L1 antibodies. NAP1L1 and LANA bound chromatin at TR was quantified by real-time PCR (qRT-PCR), using a specific primer set for the TR element. Relative binding of NAP1L1 showed reduced binding in LANA depleted BCBL-1 (Aa) and BC-3 (Ba) cells as compared to the control cells. The error bars represent standard deviations of the means from at least three experimental replicates. The *P* values were calculated by two-tailed t tests comparing LANA and NAP1L1 depleted cells with control cells by using Prism 6.0 software. Asterisk indicates significance, *P < 0.05; **P < 0.01; ***P < 0.001; ****P < 0.0001. **(C)** NAP1L1 depleted cells showed reduced nucleosome on viral DNA. NAP1L1 depleted or control BCBL-1 and BC-3 cells were harvested for MNase digestion. Nuclei were digested with 50 U of MNase before extracting the DNA for the detection of nucleosome occupied DNA. NAP1L1 depleted BCBL-1 and BC-3 cells showed reduced nucleosome occupancy evidenced by smearing (lanes 2 and 4) as compared to the control cells (lanes 1 and 3). NAP1L1 depleted HEK 293L cells were transfected with TR plasmid along with LANA expressing plasmid to test nucleosome occupancy on the newly synthesized DNA. Cells depleted for NAP1L1 showed complete digestion with MNase (lane 6) as compared to the control cells (lane 5), which showed a ladder of MNase resistant bands confirming nucleosome occupancy.

LANA binds within the TR region of the KSHV genome along with various cellular proteins that are important for DNA replication. Since NAP1L1 was important for DNA replication, we wanted to determine whether NAP1L1 associated with the TR region. To this end, we performed chromatin immunoprecipitation assays (ChIP) with NAP1L1 from BCBL-1 (Fig. 6A) and BC-3 (Fig. 6B) cells to determine its binding at the TR. This showed the reduced binding of NAP1L1 in LANA-depleted cells as compared to the control cells (Fig. 6Aa,Ba). This confirmed that NAP1L1 is associated with the terminal repeat region through LANA. NAP1L1 binding to the terminal repeat region was significantly reduced in NAP1L1-depleted cells, as expected (Fig. 6Aa,Ba). We also tested the relative binding of LANA in NAP1L1-depleted cells to determine whether the nucleosome assembly protein affected the binding of LANA to the TR chromatin. To our surprise, we saw a slight increase in LANA-bound chromatin in NAP1L1-depleted cells as compared to the control cells (Fig. 6Aa,Ba). These results suggested that LANA recruits NAP1L1 to the terminal repeat region, possibly for assembling the nucleosome following LANA-mediated replication.

### NAP1L1-depleted cells showed reduced nucleosome on the KSHV genome

The nucleosome assembly during DNA replication is a critical step in packaging newly synthesized DNA into nucleosomal arrays by the nucleosome assembly factors^39^,^46^. Based on the association of NAP1L1 with LANA, we wanted to determine whether NAP1L1 plays a functional role in assembling the nucleosome following replication of the KSHV genome *in vivo.* KSHV episome attaches to the chromosome via LANA binding within the TR element, which also initiates DNA replication, therefore we determined the chromatin structure on the TR DNA by micrococcal nuclease (MNase) digestion of viral chromatin from KSHV-infected BCBL-1 and BC-3 cells. Nuclei isolated from BCBL-1 and BC-3 cells depleted for NAP1L1 or control cells were subjected to MNase digestion for the detection of nucleosome-occupied DNA by extracting DNA from the MNase-digested chromatin and hybridizing with a TR probe (Fig. 6C). DNA extracted from the MNase-digested shControl (shCon) BCBL-1 and BC-3 cells showed a pattern of nucleosome-assembled, MNase-digested, DNA (Fig. 6C, lanes 1 and 3). The 147 bp DNA is wrapped around the mononucleosome and bands in the multimer of 147 bp are indicative of a well-packed nucleosome. The DNA from NAP1L1-depleted, BCBL-1 and BC-3 cells showed a smear or less defined pattern suggesting a lack of proper nucleosome positioning, and thus provided access to the micrococcal nuclease digestion (Fig. 6C, lanes 2 and 4). This confirmed that NAP1L1 helps in assembling the chromatin on the KSHV genome.

Furthermore, we tested the role of NAP1L1 in nucleosome assembly on transiently transfected TR-containing plasmid in NAP1L1-depleted cells. NAP1L1-depleted or control HEK 293L cells were transfected with TR plasmid along with LANA expression vector for the isolation of nuclei 4 days post-infection. DNA from the MNase-digested nuclei of control cells (shCon) showed a ladder of bands as compared to a single undefined band in NAP1L1-depleted cells (Fig. 6C, compare lanes 5 and 6). This confirmed that a lack of NAP1L1 causes decreased in nucleosome assembly post-replication.

### NAP1L1 was present at the replication fork similar to LANA

**Figure 7:**
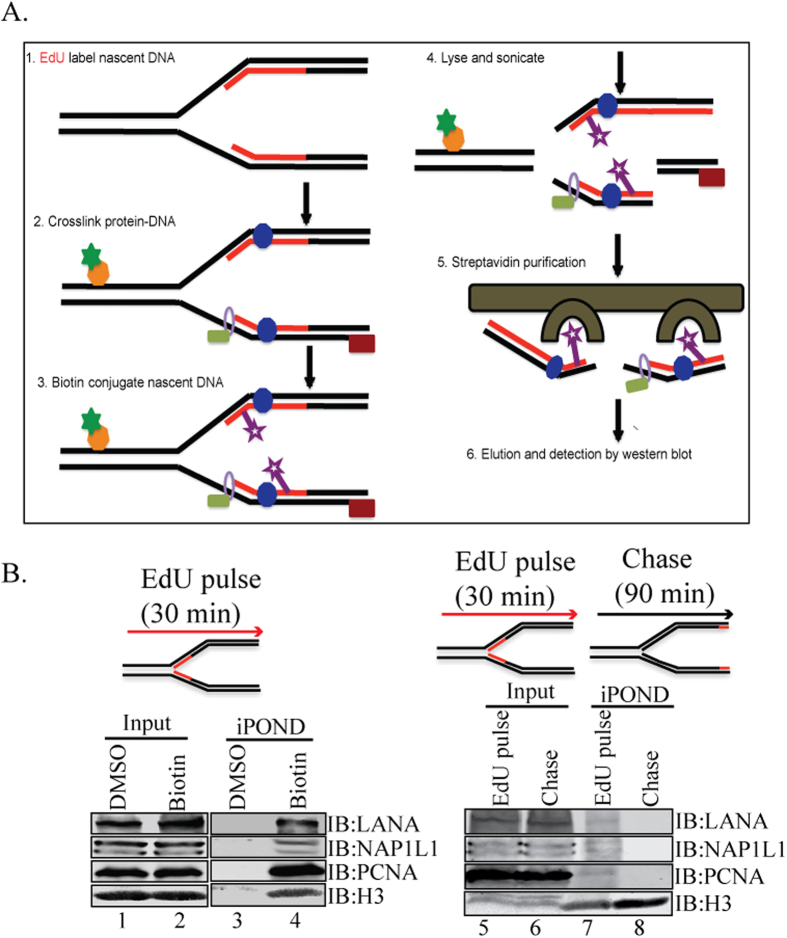
NAP1L1 and LANA were detected at replication forks on replicating DNA. **(A)** An outline of the iPOND procedure. Cells are treated with EdU to label the nascent DNA *in vivo* prior to performing iPOND. The cells are then fixed with formaldehyde to cross-link protein-DNA complexes, washed and permeabilized with detergent or chase in fresh medium before fixation. EdU labeled DNA are covalently tagged with biotin using click chemistry. The cells are then lysed with sonication and the biotin-labeled protein-DNA complexes are purified using streptavidin-coated beads. **(B)** KSHV-positive cells were incubated with EdU for 30 min to label the replicating DNA for subsequent detection of NAP1L1, LANA, PCNA and histone H3 (lanes 3 and 4). Input lanes show the presence of these proteins in EdU labeled cells (lanes 1 and 2). Both, NAP1L1 and LANA were detected in EdU labeled Biotin tagged DNA (lane 4) but not in control (DMSO) sample (lane 3). PCNA and histone H3 was used as a control to determine their association with replicating DNA. EdU labeled (Pulse) cells were grown in EdU free medium for 90 min (Chase) to determine the proteins associated with replication forks. Both, LANA and NAP1L1 were detected in pulsed samples (lane 7) but not in the chased samples (lane 8). PCNA, which is part of the replication complex, was also not detected in the chased samples (lane 8). Histone H3 was detected in chased and pulsed samples as histones are the part of replicating and mature DNA (lanes 7 and 8).

DNA replication and its organization into chromatin are essential to maintain genome function and integrity. Replication-coupled nucleosome assembly is the first step in chromatin restoration that takes place immediately behind the replication fork through the recruitment of a number of histone chaperones, such as NAP1L1, CAF-1, the replicative clamp PCNA and the clamp loader RFC^47^,^48^. We used a recently developed biochemical method, isolation of proteins on nascent DNA (iPOND) that enables the purification of proteins associated with nascent DNA at replication forks or on chromatin following DNA replication^49^,^50^. The iPOND method relies on EdU labeling of the replicating DNA *in vivo*, attaching biotin to the labeled EdU (clicking) and affinity purification of the bound proteins following analysis using standard protein detection methods (Fig. 7A). We labeled the KSHV-positive cells with a nucleoside analog, 5-ethynyl-2′-deoxyuridine, for 30 min followed by fixing the protein DNA complex to determine the proteins associated with newly synthesized DNA. The chromatin isolated from these labeled cells was covalently conjugated with Biotin or DMSO as control. The biotin-labeled, newly replicated DNA and the bound proteins were affinity purified and used for the detection of NAP1L1 and LANA. Presence of NAP1L1 and LANA in the biotin-conjugated sample but not in the DMSO sample suggested that these two proteins were present together in the replicating DNA (Fig. 7B, compare lanes 3 and 4). Detection of histone H3 and PCNA in the replicated DNA, which has been shown to be present on replicated DNA, confirmed the specificity of the assay^49^,^50^. Further, we wanted to determine whether NAP1L1 is present on the replicated DNA or moves along with the replication fork. This was determined by allowing the EdU-labeled cells to grow in the absence of EdU for 90 min (nucleotide chase). This allows the isolation of EdU-labeled mature chromatin post-replication but lacks the proteins of replication forks due to the chase. As shown in Fig. 7B, histone H3 was detected in both pulsed and chased samples, thus confirming that histone H3 is present on the mature chromatin post-replication (Fig. 7B, lanes 7 and 8). PCNA, which is part of the replication complex, was detected in the pulsed, but not in the chased sample, which confirmed its movement along with the replication forks as shown previously (Fig. 7B, compare lanes 7 and 8)^50^,^51^. Detection of NAP1L1 and LANA in pulsed samples only suggested that these proteins are part of the replicative and post-replicative complex (Fig. 7B, compare lanes 7 and 8).

### Depletion of NAP1L1 up regulated viral genes expression

**Figure 8:**
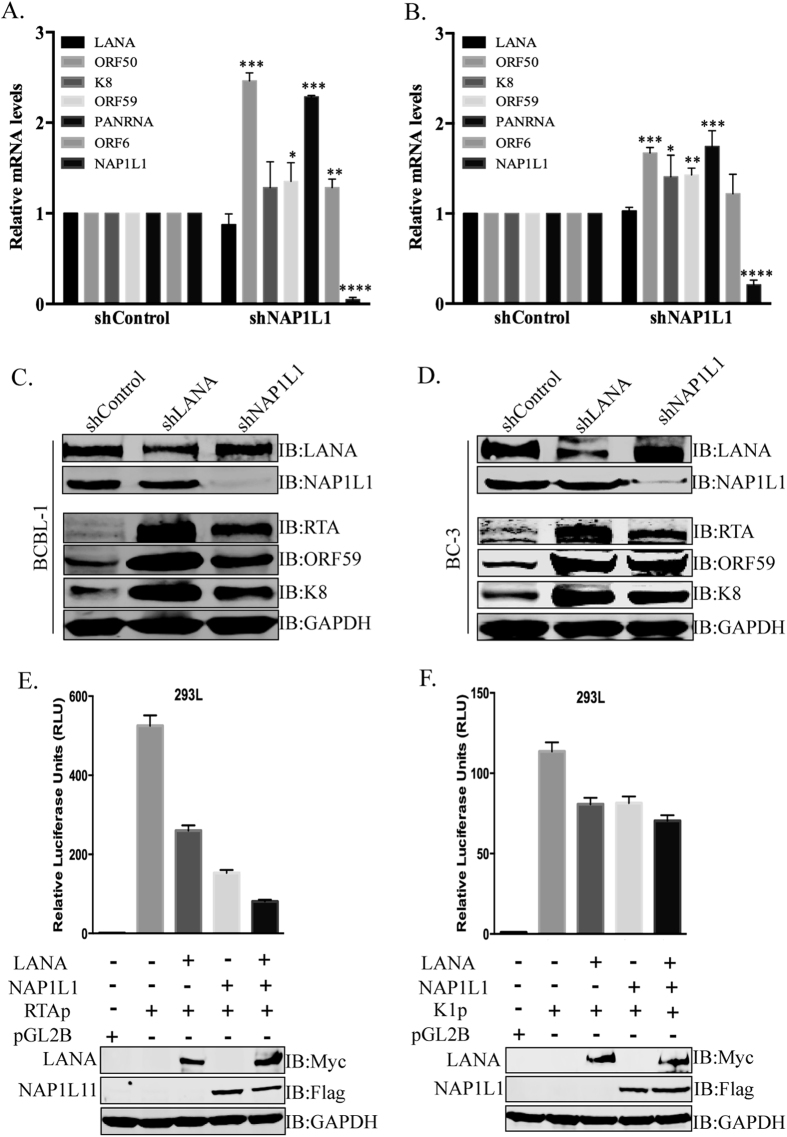
NAP1L1 depletion altered viral gene expression and regulated transcriptional activity. Relative mRNA levels of viral lytic genes in NAP1L1 depleted cells as compared to the control (shControl) BCBL-1 **(A)** and BC-3 **(B)** cells. Total RNA extracted from these cells was subjected to qRT-PCR using KSHV-specific primer for the quantitation of lytic genes along with latent gene, LANA. Depletion of NAP1L1 triggered the transcription of lytic genes. The error bars represent the standard deviations from the mean of at least three independent experiments. The *P* values were calculated by two-tailed t tests comparing NAP1L1 depleted cells with control cells. Statistical analysis was performed using Prism 6.0 software. Asterisks indicates significance, *P < 0.05; **P < 0.01; ***P < 0.001; ****P < 0.0001. **(C**,**D)** Expression profiles of the viral genes in NAP1L1 depleted, LANA depleted and control BCBL-1 **(C)** and BC-3 **(D)** cells. Depletion of LANA (shLANA lane) upregulated the expression of lytic proteins, RTA, ORF59 and K8 as compared to the control cells (shControl). Depeltion of NAP1L1 (shNAP1L1 lane) was also able to upregulate the expressions of these lytic proteins as compared to the control (shControl). GAPDH was used as loading control. **(E**,**F)** NAP1L1 regulated the transcription of RTA **(E)** and K1 **(F)** promoters. The RTA (RTAp) and K1 (K1p) promoters fused to luciferase reporter was transiently transfected into HEK 293L cells with LANA, NAP1L1 individually or together. Both, NAP1L1 and LANA were able to repress the transcriptional acivities of these promoters and showed syngistic effect when expressed together. The error bars represent standard deviations of the means from three independent experiments. Protein lysates were analyzed by the Western blots for the detection of transfected protein with anti-Myc for LANA and anti-Flag for NAP1L1. The GAPDH immunoblot shows equal loading of the cell lysates.

LANA is essential for the replication and persistence of the viral episome during latent infection^7^,^24^. LANA also negatively regulates the transcription of viral lytic genes and helps in the establishment of latency^17^,^19^. Depletion of LANA results in the loss of the viral genome and enhanced lytic gene expression^17^. In this study, we found that LANA recruits NAP1L1 for the assembly of the nucleosome and therefore postulated on whether depletion of NAP1L1 affects nucleosome structure for gene activation. We used NAP1L1-depleted, KSHV-infected, BCBL-1 and BC-3 cells for the detection of KSHV gene activation as compared to the control cells. Relative quantitation of the viral genes showed an increase in the levels of immediate early gene, RTA and PAN RNA, suggesting the involvement of NAP1L1 in regulating the chromatin on these promoters (Fig. 8A,B). Expression of early and late genes, ORF59, K8, and ORF6, respectively, were also slightly enhanced in NAP1L1-depleted cells. The levels of NAP1L1 were significantly reduced, confirming an efficient knockdown of NAP1L1 in shRNA-expressing cells (Fig. 8A,B). Expression of LANA was unchanged, possibly because the LANA promoter remains active during latent infection. These results indicate that LANA-recruited NAP1L1 is crucial for the maintenance of latent infection. We further determined the effect of NAP1L1 depletion on the expressions of viral genes LANA, RTA, K8 and ORF59 in a Western blot (Fig. 8C,D). We found enhanced expressions of KSHV lytic proteins in NAP1L1-depleted cells as compared to control cells in both the cell lines, BCBL-1 and BC-3 (Fig. 8C,D). Depletion of LANA robustly enhanced the expression of lytic genes, as expected (Fig. 8C,D). The relative levels of lytic genes expression were significantly higher in LANA-depleted cells but the depletion of NAP1L1 also resulted in increased levels of these proteins when compared to the control cells. This confirmed that NAP1L1 plays a role in regulating the viral chromatin.

### NAP1L1 regulated transcription of RTA and K1 promoters

Since we identified NAP1L1 as the LANA binding protein, and observed that its depletion resulted in an increased expression of viral genes, we wanted to determine whether NAP1L1 could directly affect the transcription of viral promoters. To this end, we used two well-characterized LANA-regulated promoters, K1 and RTA, in a luciferase reporter assay^12^,^19^. HEK 293L cells were cotransfected with Renilla luciferase reporter plasmid (for normalization) and firefly luciferase regulated by a RTA and K1 promoter sequence along with NAP1L1 and LANA expression vectors. The data presented as relative luciferase units (RLU) showed significantly reduced RTA promoter (RTAp) activity when NAP1L1 was expressed with LANA (Fig. 8E). K1 promoter (K1p) also showed a slight reduction in the promoter activity when LANA was co-expressed with NAP1L1 as compared to the cells expressing these proteins individually (Fig. 8F). These data confirmed the importance of LANA-recruited NAP1L1 in regulating the transcriptional activities of RTA and K1 promoters. Cell lysates were analyzed for the expression of transfected proteins with anti-Myc for LANA and anti-Flag for NAP1L1. GAPDH was used as the loading control.

## Discussion

KSHV is closely linked to multiple human malignancies, including Kaposi’s sarcoma (KS), primary effusion lymphomas (PELs) and multicentric Castleman’s disease^1^,^2^. KSHV establishes a lifelong latent infection with the expression of a limited number of viral genes^4^. LANA is among the most predominantly expressed protein, which helps in tethering the viral genome to the host chromosome^3^,^7^. LANA regulates transcriptional control as well as episomal DNA replication by recruiting various cellular proteins^52^. Binding of LANA within the TR region helps in the initiation of latent DNA replication in the TR as well as the segregation of replicated episomes during cell division^8^,^24^,^27^.

Multiple proteins were identified as LANA interacting proteins in our 2D-DIGE assay. These included proteins involved in transcriptional regulation, DNA replication, posttranslational modification, nucleosome assembly and the histones H2A and H2B. Some of these proteins were previously identified as LANA binding proteins^10^. In this study, we characterized the interaction of a nucleosome assembly protein, NAP1L1, with LANA. NAP1L1 acts as a histone chaperone, or a nucleocytoplasmic shuttling protein that is crucial for the movement of histones into the nucleus^44^. NAP1L1 is a member of the NAP family, which is involved in histone-histone and histone-DNA interactions involving replication, transcription and chromatin assembly^44^. This family of proteins helps in chromatin assembly^53^ and in the regulation of cell-cycle progression^41^. We confirmed the interaction of NAP1L1 with LANA in multiple assays, which showed a strong association and that it was localized in the same nuclear compartments of the KSHV-infected cells.

We identified the domain of LANA responsible for its interactions with NAP1L1, which mapped to the N-terminal domain. The amino-terminal domain of LANA is known to associate with the host chromatin through the chromatin-binding domain^9^ and the NAP1L1 binding region was identified in close proximity to this chromatin-binding region. The N-terminal domain of LANA associates with histones for tethering to the host chromosome required for replication and episome persistence^3^,^9^. A binding analysis with bacterially expressed protein confirmed the direct interaction of LANA-N with NAP1L1. The carboxy terminal domain of LANA binds directly to the LANA-binding sites (LBS) of TR and helps in initiating DNA replication^3^,^11^,^52^. Additionally, the amino and carboxy terminal domains of LANA associate with each other^10^, therefore, it could be assumed that the close proximity of LANA-recruited NAP1L1 helps in the assembly of nucleosomes on the newly replicated DNA.

NAP1L1 consists of 391 amino acid proteins with three acidic domains, a leucine-rich nuclear export sequence, a long N-terminal sequence with a conserved NAP domain, and the C-terminal acidic domain^36^,^39^. Among these, the C-terminal region is highly conserved in other members of the NAP-family of histone chaperones, including TAF-I/SET (68% amino acid homology)^51^. Binding of LANA to NAP1L1 was mapped to the amino terminal domain of NAP1L1, which contains a series of alternate α helix/β sheet regions known to be involved in the protein-protein interaction^36^, thus confirming the specificity of their interaction. The interaction of these two proteins was confirmed to be in the same nuclear compartments by their localization assays. NAP1L1 is a nucleocytoplasmic shuttling protein and conserved NES resides within the dimerization domain, which determines its cytoplasmic-nuclear distribution^54^. Previous work has demonstrated that NAP1 localizes to the nucleus in a cell-cycle dependent manner, mostly in the nucleus during the S-phase and cytoplasmic during the G_2_ phase^39^. Consistent with this, LANA associated with NAP1L1 mostly during the S-phase because the viral genome replicates during the S-phase.

Functional significance of this interaction was assayed by analyzing replication, plasmid maintenance and nucleosome occupancy on the KSHV genome. Since NAP1L1 helps in assembling the nucleosome after replication, we assumed that LANA recruits NAP1L1 to assist the proper assembly of the nucleosome on the replicated viral DNA. The transient replication assay with an excess of NAP1L1 enhanced the replication of TR plasmids and depletion repressed the replication efficiency, which thus confirms the importance of NAP1L1 in DNA replication. In addition, NAP1L1-depleted cells maintained lower copies of the TR plasmid in LANA-expressing cells, thereby confirming the necessity of chromatin assembly in plasmid maintenance. Stable plasmid maintenance requires both replication and segregation and LANA has been shown to be involved in the segregation of the replicated KSHV genome^55^, therefore the reduction in the copies of the maintained plasmids was due to the lack of proper chromatinization post-replication. The role of NAP1L1 in the maintenance of KSHV episomes was further established by the depletion of NAP1L1 in the KSHV-infected, BCBL-1 and BC-3 cells, which showed a reduction in the episomal copies of the KSHV genome. Less copies of the episomal DNA in NAP1L1-depleted cells was most likely due to the lack of the proper assembly of nucleosomes to package them in a highly ordered chromatin.

Mapping of protein binding sites on DNA in chromatin immunoprecipitation assays (ChIP) usually requires the detection of the target sequence in the immunoprecipitated DNA. The association of NAP1L1 to the viral chromatin was determined in a ChIP assay, which confirmed that LANA facilitates the recruitment of NAP1L1 to the TR region of KSHV. This was evident as the presence of LANA enhanced the binding of NAP1L1 and the depletion of LANA repressed the NAP1L1’s binding to the TR chromatin. Recent studies on Epstein-Barr virus (EBV) revealed that NAP1 assembles at the FR and DS element of *oriP*^56^; this may suggest that these two gammaherpesviruses use a similar mechanism to replicate and chromatinize post-replication. Our ChIP data of LANA binding on the TR in the absence of NAP1L1 showed that the slightly enhanced binding of LANA may be due to the steric effect of NAP1L1; or, it could be due to a change in the protein complex with LANA at the TR. Importantly, the localization of NAP1L1 on TR in cooperation with LANA to influence genome replication and persistence confirms its role in viral genome maintenance. Furthermore, it would be interesting to evaluate the interactions of these two proteins to regulate the chromatin assembly on cellular genome as LANA has been shown to associate to the host’s cellular DNA^57^.

NAP1L1 is a chromatin-associated protein and promotes nucleosome assembly leading to a compact chromatin structure^53^. Assembly of chromatin is coupled with DNA replication and an *in vitro* chromatin assembly assay led to the identification of CAF-1 and the loaded PCNA as the key components of replication-coupled chromatin assembly^58^,^59^. In addition, histone chaperones such as NAP1 and FACT also participate in replication-coupled chromatin assembly^39^,^60^. An association of NAP1L1 with LANA at the TR led us to determine the chromatin structure by digesting the chromatin of KSHV-infected cells lacking NAP1L1 with MNase that can generate mononucleosomes. Genomic regions with highly compact chromatin are not digested to yield mononucleosomes, whereas the region with less compact chromatin yields mononucleosomes^39^,^46^. Micrococcal nuclease digestion performed in presence of LANA at the TR region revealed that nucleosomes were organized in a highly ordered chromatin in cells with NAP1L1 confirming its role in chromatinization of the viral genome. Additionally, the HEK 293L cells depleted for NAP1L1 were unable to package the replicated TR DNA into higher order chromatin following replication as the chromatin digested with MNase produced only the mononucleosome. This confirms that NAP1L1 promotes nucleosome assembly and participates in replication-coupled chromatin assembly *in vivo*^39^,^46^,^60^.

We also showed that NAP1L1 is part of the replication machinery by analyzing the proteins associated with nascently synthesized DNA in an iPOND assay, which refers to the isolation of proteins on nascent DNA^50^. This assay allows for the identification of chromatin-bound proteins associating with nascent DNA by labeling the DNA with nucleotide analog, EdU. Furthermore, combining iPOND with pulse and chase methods provides a high-resolution spatiotemporal analysis of protein dynamics at replication forks. Moreover, iPOND also facilitates an analysis of posttranslational modifications and DNA excision repairs. When coupled with mass spectrometry, iPOND facilitates studies of chromatin assembly and maturation during DNA replication^50^. An analysis of proteins from the iPOND assay for NAP1L1 confirmed its association with the nascent DNA in complex with other chromatin-associated proteins, including histones and PCNA. Absence of NAP1L1 on the chased DNA confirmed that these proteins move along with replication forks and we presume that NAP1L1 is part of the post-replication chromatin assembly component.

In herpesviruses, chromatinization of the viral genome aids in establishing latency, which is characterized by silencing the expression of lytic genes^61^. Our results demonstrated that depletion of NAP1L1 in KSHV-infected cells led to the expression of viral genes capable of inducing the cascade of lytic reactivation. This expression may be due to a lack of compact chromatinization coupled with epigenetic modification of the histones. Nucleosome assembly protein can form a complex with histone methyltransferases and deacetylase affecting histone methylation and acetylation to regulate gene expression^62^. Previous studies have demonstrated that the transcriptional repression of promoters by LANA can be interrupted by deacetylase inhibitors^18^. Therefore, the epigenetic modification indicates that the interaction between LANA and nucleosome assembly protein could be important for the latent KSHV genome to maintain a hypoacetylated state.

In addition to the nucleosome assembly and histone binding activity, nucleosome assembly proteins are implicated in transcriptional regulation^42^. NAP1L1 can serve as a transcriptional activator as well as a repressor. We revealed that NAP1L1 was involved in transcriptional repression by LANA in a reporter gene assay. Overexpression of the nucleosome assembly proteins along with LANA resulted in a decreased transcriptional activity of the RTA and K1 genes’ promoters. This may be because of the squelching mechanism, where the formation of a particular complex containing the nucleosome assembly proteins is disrupted. Similar observations were made with *Xenopus* NAP1, as an overexpression of this protein in *Xenopus laevis* embryos resulted in embryonic defects^63^. Further, nucleosome assembly proteins are known to mediate interactions between p300 (coactivator) and transcription factors, such as p53 and the cell cycle-regulating transcription factor, E2F, which can form a ternary complex^42^,^64^. Therefore, we conclude that NAP1L1 controls transcriptional activity, most likely at the level of chromatin assembly^65^ and by regulating the multi-complex interaction of coactivator p300 with histones. In conclusion, this work indicates the importance of the LANA-mediated recruitment of nucleosome assembly protein NAP1L1 at the TR element of the KSHV episome. They contribute to a replication-coupled nucleosome assembly and transcriptional regulation, thus indicating that the nucleosome assembly proteins also play important roles in KSHV latent infection. Therefore, the identification of the relevant cellular and viral proteins involved in the regulation of the chromatin structure of gammaherpesviruses can lead to the development of new therapeutic targets for controlling KSHV latent infection and pathogenesis.

## Methods

### Cell culture

The KSHV-negative, Burkitt lymphoma cell line, BJAB, KSHV-positive PEL cell lines, BCBL-1 and BC-3 were cultured in RPMI 1640 medium supplemented with 10% fetal bovine serum, 2 mM L-glutamine, and penicillin-streptomycin (5 U/ml and 5 μg/ml, respectively). The human embryonic kidney cell lines, HEK 293T and HEK 293L were cultured in Dulbecco’s modified Eagle’s medium (DMEM) supplemented with 10% fetal bovine serum, 2 mM L-glutamine, and penicillin-streptomycin (5 U/ml and 5 μg/ml, respectively). All cell lines were grown at 37 °C in a humidified environment with 5% CO_2_. Deidentified human cells were used in these assays and all the experiments were done in accordance with guidelines of the University of Nevada, Reno. The Environmental and Biological Safety committee of the University of Nevada, Reno, approved the methods and techniques used in this study.

### Antibodies

The following commercial antibodies were used for this study: rat anti-LANA (Advanced Biotechnologies, Inc.), mouse anti-GAPDH (US Biological), mouse anti-Flag M2 (Sigma-Aldrich, St. Louis, MO, USA), mouse anti-Myc 9E10 (Sigma-Aldrich, St. Louis, MO, USA), rabbit polyclonal anti-NAP1L1 (Santa Cruz Biotechnology), mouse monoclonal anti-NAP1L1 (Santa Cruz Biotechnology). Mouse monoclonal anti-LANA antibody was generated at GenScript. Mouse anti-ORF59 and rabbit anti-RTA antibodies were generously provided by Dr. Bala Chandran, Rosalind Franklin University of Medicine and Science, Chicago and Dr. Yoshihiro Izumiya, UC Davis, respectively. Mouse anti-K8 antibody was obtained from Dr. Rossetto’s laboratory (University of Nevada, Reno, NV).

### Plasmids

Full-length NAP1L1 and its deletion constructs were generated by PCR amplification and cloning into a Flag-tagged vector: pA3F. Integrity of the clones was confirmed by sequencing at the Nevada Genomics Center, University of Nevada, Reno, NV. Flag-tagged LANA, pA3F-LANA and its deletion constructs, LANA-N terminal domain (1 to 340 aa) and LANA-C terminal domain (940 to 1162 aa), Myc-tagged LANA, pA3M-LANA, GFP-NLS-Myc, GFP–LANA-N-Myc (1 to 340 aa) and the truncation mutants GFP–LANA-N250-Myc (1 to 250 aa), GFP–LANA-N150-Myc (1 to 150 aa), GFP–LANA-N32-Myc (1 to 32 aa) and GFP–LANA-N33–150-Myc (33 to 150 aa) were described earlier^66^,^67^. Short hairpin RNA (shRNA)-expressing plasmids, control shRNA (shControl) and LANA shRNA (shLANA) were described previously^14^. The shRNA vectors for NAP1L1 (shNAP1L1) were purchased from GE healthcare.

### Transduction of lentiviral vectors for shRNA mediated knockdown

The pGIPz lentiviral vector (Dharmacon GE Life Sciences) containing shRNA for NAP1L1 and LANA were cotransfected with lentivrus packaging vectors, pCMV-dR8.2 and pCMV-VSVG (Addgene, Inc., Cambridge, MA) into HEK 293T cells using polyethylenimine (PEI) (Polysciences, Inc.) to produce the respective lentiviral particles. Supernatants from HEK 293T cells transfected with the shRNA and packaging vectors were collected for 5 days, followed by concentration of the virus by ultracentrifugation (25,000 rpm, 1.5 hr, 4 °C). The concentrated lentiviral particles were used for transducing the target cells (BCBL-1, BC-3 and HEK 293L) in presence of 5 μg/ml polybrene followed by selection with 1 μg/ml puromycin for pure population of cells. The RNA interference (RNAi) efficiency was assessed by Western blot analysis with specific NAP1L1 and LANA antibodies.

### Two-dimensional difference gel electrophoresis (2D-DIGE)

BCBL-1 cells were lysed in RIPA cell lysis buffer (50 mM Tris-HCl, pH 7.5, 150 mM NaCl, 1 mM EDTA and 1% NP-40) supplemented with protease inhibitors (1 mM phenylmethylsulfonyl fluoride, 10 μg/ml pepstatin, 10 μg/ml leupeptin and 10 μg/ml aprotinin) for immunoprecipitation with anti-LANA and anti-Rat IgG isogenic control antibodies. The immunoprecipitated complexes were sent to Applied Biomics (Hayward, CA) for the identification of LANA-bound proteins in a 2D-DIGE assay. Briefly, proteins precipitated with anti-LANA and control, anti-rat IgG antibodies were labeled with Cy3 and Cy5, respectively before multiplexing to resolve on a single gel. Upon completion of the first-dimension IEF, the second dimension proteins separation was performed on DIGE gels. Image of the combined Cy3 and Cy5 labeled proteins was scanned and the unique spots, compared to the control, were picked for in-gel digestion for MALDI-TOF analysis. Ten to twenty most abundant peptides in each sample were further subjected to fragmentation and tandem mass spectrometry (MS/MS analysis). Combined MS and MS/MS spectra were used for database search using GPS Explorer software equipped with the MASCOT search engine (Applied Biomics, Inc. Hayward, CA) to identify proteins from primary sequence databases.

### Co-immunoprecipitation assays

Approximately 20 million cells expressing the proteins of interest were washed with PBS (phosphate buffered saline, 10 mM NaPO4, 137 mM NaCl, 2.5 mM KCl, pH 7.5) and lysed in RIPA cell lysis buffer (50 mM Tris-HCl, pH 7.5, 150 mM NaCl, 1 mM EDTA and 1% NP-40) supplemented with protease inhibitors (1 mM phenylmethylsulfonyl fluoride, 10 μg/ml pepstatin, 10 μg/ml leupeptin and 10 μg/ml aprotinin). Cellular lysates were sonicated to shear the DNA and centrifuged at 12,000 rpm for 10 min at 4 °C to remove cellular debris. The supernatants were pre-cleared with protein A + G sepharose beads (GE Healthcare) for 30 min at 4 °C and rotated overnight with specific antibodies. Resulting immunocomplexes were captured with protein A + G conjugated sepharose beads by rotating them for 2 hr at 4 °C. The immunocomplexes were collected by centrifugation at 2000 rpm for 2 min at 4 °C. The beads were washed three times with 1 ml of ice-cold RIPA buffer supplemented with protease inhibitors and boiled in 50 μl of SDS PAGE sample loading buffer for 5 min. The immunoprecipitated proteins and respective total cell lysates were resolved on SDS-polyacrylamide gel and transferred onto 0.45-μm nitrocellulose membranes (GE Healthcare) at 100 V for 80 min. The blots were blocked with 5% non-fat milk in TBST buffer (10 mM Tris-HCl, pH 7.5, 150 mM NaCl, 0.05% Tween 20) and washed three times with TBST buffer before incubating overnight at 4 °C with specific primary antibodies. The blots were washed three times with TBST followed by incubating with appropriate secondary antibodies conjugated with AlexaFluor 680 or AlexaFluor 800 (Molecular Probes, Carlsbad, CA) and secondary antibodies at 1:10,000 dilutions. The membranes were scanned with an Odyssey infrared scanner (LI- COR Biosciences, Lincoln, NE).

### Immunofluorescence assay (IFA)

KSHV-positive BCBL-1 and BC-3 cells were washed with phosphate-buffered saline (PBS) before spreading on coverslips. The cells were allowed to air-dry for 10 min, fixed with 4% paraformaldehyde for 10 min at room temperature and followed by permeabilization with 0.2% Triton X-100 in PBS for 10 min at room temperature. Cells were blocked with PBS containing 0.4% fish skin gelatin and 0.05% Triton X-100 for 30 min at room temperature. The cells were then incubated with specific primary antibodies for 1 hr at room temperature and washed with PBS before being incubated with AlexaFluor conjugated secondary antibodies (Molecular Probes) for 45 min at room temperature. The cells were washed three times with PBS to remove non-specifically bound antibodies before staining with nuclear stain, TO-PRO-3 (Molecular Probes). Images were captured using a confocal laser-scanning microscope (Carl Zeiss, Inc.).

An immunofluorescence assay on the KSHV-positive cells arrested in the G_2_/M phase was done after treating them with colchicines. Cells were grown on coverslips coated with poly-L-lysine followed by incubation with colchicine for 120 min before fixing with formaldehyde. Staining and microscopy were performed as described above.

### Dual luciferase reporter assay

HEK 293L cells were seeded into 6-well plates the day before transfection. The cells were co-transfected using Metafectene (Biontex Laboratories, GmbH) with RTA and K1-Luc reporter plasmids (pGL2 containing full-length RTA and K1 promoter) and the plasmids expressing LANA and NAP1L1. Transfection efficiencies were monitored by transfection of the GFP-containing vector, pEGFP. The Renilla luciferase-expressing plasmid (pRRLSV40) was transfected for data normalization in a dual reporter assay. At 24 h post-transfection, the cells were lysed in cell lysis buffer (Promega, WI) and 50 μl of this lysate was used for the reporter assay in a dual-luciferase reporter assay (Promega, WI). Luminescence was measured using the Chameleon luminometer (Hidex). A portion of the cell lysates was used for Western blotting to detect LANA, NAP1L and GAPDH. All experiments were performed in triplicate, and the results shown represent the means of three independent experiments.

### Cell viability-MTT assay

The Vibrant MTT cell proliferation assay kit (Life Technologies, Inc. Grand Island, NY, USA) was used for cell viability assay according to the manufacturer’s instruction. Briefly, KSHV-positive, BCBL-1 and BC-3 cells depleted for LANA and NAP1L1, and HEK 293L cells depleted for NAP1L1 (shNAP1L1) and control (shCon) cells were grown in RPMI 1640 and DMEM medium without phenol red. Approximately, 100,000 cells were transferred to each well in a 96 well microplate. A total of 10 μl of the 12 mM MTT (3-[4,5-dimethyl-2- thiazolyl]-2,5-diphenyl tetrazolium bromide) stock solution was added to each well. One hundred microliters of medium alone was used as negative control. Cells were incubated at 37 °C for 4 h followed by addition of 100 μl of the SDS-HCl solution. The microplate was then incubated at 37 °C for another 4 h and the absorbance was recorded at 570 nm. Assays were performed in triplicate.

### Transient DNA replication assay

For replication assays involving overexpression and protein silencing by shRNA, HEK 293L cells in 100-mm dishes were co-transfected with 20 μg of TR-containing plasmid with 20 μg of plasmids overexpressing LANA, NAP1L1 or with an empty vector, pA3F/pA3M, as filler DNA. Similarly, HEK 293L cells depleted for NAP1L1 were transfected with 20 μg KSHV TR-containing plasmid with 20 μg of KSHV LANA expression plasmid. At 96 h post-transfection, cells were collected and washed twice with phosphate-buffered saline followed by extraction of DNA using a modified Hirt’s lysis method, described earlier^68^. Extracted DNA was dissolved in 50 μl of distilled water containing RNase. Ten percent of the extracted DNA was linearized with *Eco*RI and the remainder with *Dpn*I and *Eco*RI to remove the non-replicated DNA. The digested DNA was separated on 0.8% agarose gel followed by Southern transfer onto a Hybond N+ membrane (GE Healthcare) to hybridize with ^32^P-labeled TR probes. Probes specific to the KSHV TR were synthesized using a random primer labeling kit followed by purification on G-50 columns (GE Healthcare). The auto radiographic signals were detected using a PhosphorImager, according to the manufacturer’s instructions (Molecular Dynamics, Inc.). Signals were quantified using ImageQuant software (Molecular Dynamics, Inc.). Replicated DNA was determined by analyzing the relative densities of the *Dpn*I-resistant bands and normalizing them with the respective *Eco*RI bands in the input lane.

### Plasmid maintenance assay

HEK 293L cells depleted for NAP1L1 (shNAP1L1) and control cells (shControl) in 100-mm dishes were co-transfected with 20 μg of KSHV TR-containing plasmid with 20 μg of KSHV LANA expression plasmid. Cells were harvested 6 h post-transfection and replated onto 100-mm dishes to grow for 7 days, followed by an extraction of the DNA from 5 × 10^6^ cells using a modified Hirt’s method^68^. DNA was digested and linearized with *Eco*RI and analyzed by Southern blotting as described for transient replication assays. Linearized plasmid bands were visualized by autoradiography and quantified using PhosphorImager and ImageQuant software (Molecular Dynamics, Inc.).

### Gardella gel analysis

Gardella gels were used to assess the episome maintenance^69^. KSHV-positive BCBL-1 and BC-3 cells depleted for LANA and NAP1L1 were loaded into the agarose gel with a lysis plug containing DNase-free Proteinase K (Sigma-Aldrich, St. Louis, MO, USA) and SDS followed by electrophoresis in a Tris-borate-EDTA buffer. The gels were run overnight at 15 V and then at 110 V for another 24 h. DNA was transferred to a Hybond N + membrane (GE Healthcare) and hybridized with ^32^P-labeled TR probes to detect KSHV episome. Relative KSHV genome copies were analyzed by real-time PCR using a KSHV TR-specific primer. Each assay was performed in triplicate and the data shown are the means of three experiments.

### MNase protection assay

Cells were collected by centrifugation and resuspended in 1.5 ml of Buffer A (300 mM sucrose, 35-mM Tris-HCl, pH 7.9, 15 mM NaCl, 5 mM MgCl_2_, 60 mM KCl, 3 mM CaCl_2_, 0.2% Triton X-100) followed by incubation at 4 °C for 10 min. The pellet was collected by centrifugation at 1000xg for 15 min at 4 °C and resuspended in 0.6 ml of MNase Buffer (35 mM Tris-HCl, pH 7.9, 15 mM NaCl, 5 mM MgCl_2_, 60 mM KCl, 3 mM CaCl_2_, 12.5% glycerol) and chromatin suspension was prepared to partially digest with 50 units of micrococcal nuclease (MNase) by incubation at room temperature for 10 min with constant rotation. The reaction was stopped by adding 25 mM EDTA and 1% SDS. Following treatment with 10 μg of RNase A at 37 °C for 30 min, 50 μg of Proteinase K were added and the samples were incubated overnight at 37 °C. DNA was extracted with phenol:chloroform:isoamyl alcohol and precipitated with ethanol. The purified DNA was analyzed on 1.2% agarose gel followed by Southern transfer on Hybond N+ membrane (GE Healthcare) and hybridized with ^32^P-labeled TR probes. The signals were detected using a PhosphorImager, according to the manufacturer’s instructions (Molecular Dynamics, Inc.).

### Chromatin immunoprecipitation assay (ChIP)

Chromatin immunoprecipitation was performed as described previously^67^. Briefly, 15 to 20 million cells were fixed with a final concentration of 1% formaldehyde for 10 min at room temperature followed by the addition of glycine at a final concentration of 125 mM for 5 min to stop cross-linking. The cells were rinsed three times with ice-cold PBS and lysed in cell lysis buffer (5 mM PIPES, pH 8.0, 85 mM KCl, and 0.5 mM NP-40) supplemented with protease inhibitors for 10 min on ice. The nuclei were enriched by low-speed centrifugation and resuspended in nuclear lysis buffer (50 mM Tris-HCl, pH 8.1, 10 mM EDTA and 1% SDS) with protease inhibitors. Chromatin was sonicated to an average length of 500 to 800 bp and centrifuged for 10 min at 13,000 rpm to remove the cell debris. The resulting supernatant was diluted five-fold with ChIP dilution buffer containing 16.7 mM Tris-HCl, pH 8.1, 167 mM NaCl, 1.2 mM EDTA, 0.01% SDS, and 1.1% Triton X-100 with protease inhibitors. The diluted chromatin was pre-cleared with protein A + G sepharose beads pretreated with 1 mg/ml BSA and 1 mg/ml sheared salmon sperm DNA for 30 min at 4 °C with rotation followed by incubation overnight with either control or specific antibodies at 4 °C with rotation. Immune complexes were collected by incubating with Protein A + G sepharose beads for 1–2 h at 4 °C. The beads were collected and subsequently washed with a low-salt buffer (0.1% SDS, 1.0% Triton X-100, 2 mM EDTA, 20 mM Tris [pH 8.1], 150 mM NaCl), a high-salt buffer (0.1% SDS, 1.0% Triton X-100, 2 mM EDTA, 20 mM Tris [pH 8.1], 500 mM NaCl), and a LiCl wash buffer (0.25 M LiCl, 1.0% 269 NP-40, 1% deoxycholate, 1 mM EDTA, 10 mM Tris [pH 8.0]). The beads were then washed twice with Tris-EDTA buffer and chromatin was eluted using an elution buffer (1% SDS, 0.1 M NaHCO_3_) and reverse cross-linked by adding 0.3 M NaCl at 65 °C overnight. Eluted DNA was precipitated, treated with RNase and Proteinase K at 45 °C for 2 h and purified. Purified DNA of the ChIP fraction and the inputs were subjected to amplification of TR with the primers (forward, 5′-GGGGGACCCCGGGCAGCGAG-3′, and reverse, 5′-GGCTCCCCCAAACAGGCTCA-3′) flanking TR nucleotides 677 to 766 on an ABI StepOne plus real-time PCR machine (Applied Biosystems).

### Quantitative real-time PCR

Expression profiles of different KSHV genes in KSHV BCBL-1 and BC-3 cells depleted for NAP1L1 were measured by real-time reverse transcription (RT)-PCR. Total mRNA from the cells was extracted using an Illustra RNAspin Mini Kit (GE Healthcare) according to the manufacturer’s instructions, and cDNA was made by using a High-Capacity cDNA Reverse Transcription Kit (Applied Biosystems, USA). Each PCR reaction was set up in a total volume of 20 μl with 0.5 μM KSHV ORF-specific primers and 2 μl of cDNA. Primers for the human GAPDH (glyceraldehyde-3-phosphate dehydrogenase) housekeeping genes were included for normalizing the threshold cycle (*C*_*T*_) values. The cDNA was amplified on an ABI StepOne Plus real-time PCR machine (Applied Biosystems, USA) and the relative gene copy numbers or transcript numbers were calculated by the ∆∆*C*_*T*_ method. Each experiment included duplicate samples and the data shown represent the means of three independent experiments.

### Isolation of proteins on nascent DNA (iPOND)

KSHV-positive cells (1.0 × 10^8^ cells per sample) were incubated for 30 min with 10 μM of the thymidine analogue, EdU (5-ethynyl-2′-deoxyuridine). For chase experiments, EdU-labeled cells were washed once with a temperature and pH-equilibrated medium to remove the EdU and cells were resuspended in chase media pre-equilibrated to 37 °C and incubated for 90 min in the incubator. After pulse and chase, cells were cross-linked with 1% formaldehyde for 10 min at room temperature (RT) and quenched with 0.125 M glycine for 5 min at RT and washed three times in PBS. Cell pellets were then resuspended in 0.25% Triton-X/PBS to permeabilize and incubated for 30 min at RT. Pellets were washed once with 0.5% BSA/PBS and once with PBS using same volume as used for permeabilization prior to the click reaction.

Click reactions were performed to conjugate biotin to the EdU-labeled DNA. Cells were incubated in a click reaction buffer (Life Technologies, Inc.) for 1 h at a concentration of 1 × 10^8^ cells/ml. The click reaction buffer contained Invitrogen Click-iT cell reaction buffer and cell buffer additive (C10269), 2 mM CuSO4, and 5 μM photocleavable biotin-azide (Life Technologies, Inc.). DMSO was added instead of biotin-azide to the negative control samples. The cell pellets were washed once with 0.5% BSA/PBS and once with PBS. Cells were then resuspended in lysis buffer containing 1% SDS, 50 mM Tris (pH 8.0), 1 μg/mL leupeptin, and 1 μg/mL aprotinin and incubated on ice for 15 min. Samples were sonicated using a microtip sonicator for 10 min at 10 W with 30-sec on/off pulses. Cell debris were removed by centrifugation at 16,100 × g for 10 min at RT and diluted 1:1 (v/v) with cold PBS containing 1 μg/mL leupeptin and aprotinin.

Dynabeads MyOne Streptavidin T1 (Invitrogen) was used to capture the biotin-conjugated DNA-protein complexes. Firstly, beads were washed twice with 1:1 (v/v) of lysis buffer and once in PBS followed by incubating the samples for 16–20 h at 4 °C in the dark. The beads were centrifuged for 3 min at 1,800 × g to capture the DNA and the associated proteins. The beads were washed once with cold lysis buffer, once with 200 mM NaCl, and then twice with lysis buffer. Captured proteins were eluted under reducing conditions by boiling them in 2X SDS sample buffer for 5 min at 95 °C. Protein samples were resolved on 4–20% gradient gel (Bio-Rad Laboratories) and immunoblotted with specific antibodies using the Odyssey infrared imaging system (LI- COR Biosciences, Lincoln, NE).

### IdU labeling and immunoprecipitation of replicated DNA

NAP1L1 and LANA depleted KSHV-positive; BCBL-1 and BC-3 cells were pulsed with 30 μM of IdU (Sigma-Aldrich, St. Louis, MO, USA) for 30 min followed by collecting the cells by centrifugation after washing them twice with cold PBS. Episomal DNA, extracted by the modified Hirt’s method, was subjected for immunoprecipitation using anti-IdU antibody (BD Biosciences, Inc.). 10% of the extracted DNA was saved to use as input control. The remaining IdU labeled DNA was added with TE (10 mM Tris-HCl, 1 mM EDTA) to make 460 μl followed by addition of 40 μl of 5 mg/ml sheared and denatured salmon sperm DNA. Samples were sonicated to get an average length of 700 bp and heat denatured at 95 °C for 5 min before cooling on ice. Samples were adjusted to 10 mM sodium phosphate (pH 7.0), 140 mM NaCl and 0.05% Triton X-100 and then incubated with 1 μg of mouse anti-IdU antibody at room temperature with constant rotation for 1 h. Antibody bound IdU labeled DNA was precipitated using magnetic Protein A/G (GE Healthcare, Inc.) after incubation for 30 min. Pellets were washed three times with 750 μl of 10 mM Sod. phosphate buffer, 140 mM NaCl and 0.05% Triton X-100, resuspended in 200 μl of lysis buffer (50 mM Tris-HCl (pH8.0), 10 mM EDTA, 0.5% SDS, 0.25 mg/ml Proteinase K) and incubated overnight at 37 °C. 100 μl additional lysis buffer was added and incubated at 50 °C for 1 h. Bound DNA was purified after phenolization and precipitation for the quantitation of IdU labeled DNA in a semi-quantitative real-time PCR by amplifying the TR region.

### Statistical analysis

All statistical analyses were performed using Prism 6.0 software (GraphPad Software, Inc., CA, USA) and the P-values were calculated using two-tailed t-tests. Statistical significance is indicated by an asterisk and is described in the figure legends.

## Additional Information

**How to cite this article**: Gupta, N. *et al*. KSHV encoded LANA recruits Nucleosome Assembly Protein NAP1L1 for regulating viral DNA replication and transcription. *Sci. Rep.* **6**, 32633; doi: 10.1038/srep32633 (2016).

## Figures and Tables

**Table 1:** Identities of the unique spots in LANA immunoprecipitated samples.

Spot	Protein Name	Accession Number
1	Spectrin, alpha, non-erythrocytic 1 (alpha-fodrin),isoform CRA_b [Homo sapiens]	gi|119608212
2	nonerythroid alpha-spectrin [Homo sapiens]	gi|179106
3	ras-related protein Rab-37 isoform 3 [Homo sapiens]	gi|28376635
4	Spectrin, alpha, non-erythrocytic 1 (alpha-fodrin),isoform CRA_e [Homo sapiens]	gi|119608215
5	Alpha-actinin-4 [Homo sapiens]	gi|12025678
6	ZFYVE26 protein [Homo sapiens]	gi|28175105
7	ZFYVE26 protein [Homo sapiens]	gi|28175105
8	Brefeldin A-inhibited guanine nucleotide-exchange protein 2 OS = Homo sapiens	BIG2_HUMAN
9	ZFYVE26 protein [Homo sapiens]	gi|28175105
10	coronin, actin binding protein, 1C variant [Homo sapiens]	gi|62897707
11	Lymphocyte-specific protein 1 isoform 1 [Homo sapiens]	gi|10880979
12	vimentin [Homo sapiens]	gi|62414289
13	Heterogeneous nuclear ribonucleoprotein F [Homo sapiens]	gi|4826760
14	ARP3 actin-related protein 3 homolog variant [Homo sapiens]	gi|62088286
15	Beta actin variant [Homo sapiens]	gi|62897625
16	ras-related protein Rab-37 isoform 3 [Homo sapiens]	gi|28376635
17	ZNF483 protein [Homo sapiens]	gi|41351323
18	Eukaryotic translation elongation factor 1 gamma,isoform CRA_d [Homo sapiens]	gi|119594432
19	ras-related protein Rab-37 isoform 3 [Homo sapiens]	gi|28376635
20	F-actin capping protein alpha-1 subunit variant [Homo sapiens]	gi|62898013
21	Regulatory factor X-associated protein	GI|3287900
22	Protease serine 2 isoform B [Homo sapiens]	gi|33126583
23	hCG2002962 [Homo sapiens]	gi|119572309
24	Nucleosome assembly protein 1-like 1	gi|1709337
25	EF-hand domain-containing protein D2 [Homo sapiens]	gi|20149675
26	Capping protein (actin filament) muscle Z-line, beta [Homo sapiens]	gi|54695812
27	Regulator of microtubule dynamics protein 2 OS = Homo sapiens	RMD2_HUMAN
28	Serine/threonine-protein phosphatase 4 regulatory subunit 4 isoform 1 [Homo sapiens]	gi|17402886
29	tropomyosin alpha-3 chain isoform 2 [Homo sapiens]	gi|24119203
30	MYL6 protein [Homo sapiens]	gi|113812151
31	Protease serine 2 preproprotein [Homo sapiens]	gi|114319021
32	Glial fibrillary acidic protein isoform 2 [Homo sapiens]	gi|196115290
33	ras-related protein Rab-37 isoform 3 [Homo sapiens]	gi|28376635
34	ras-related protein Rab-37 isoform 3 [Homo sapiens]	gi|28376635
35	Histone H2B	GI|290457686
36	Histone H2A	GI|24496292
